# Effects of Grazing and Precipitation on Herbage Biomass, Herbage Nutritive Value, and Yak Performance in an Alpine Meadow on the Qinghai–Tibetan Plateau

**DOI:** 10.1371/journal.pone.0127275

**Published:** 2015-06-03

**Authors:** Fuhong Miao, Zhenggang Guo, Ran Xue, Xianzhi Wang, Yuying Shen

**Affiliations:** State Key Laboratory of Grassland Agro-ecosystems, College of Pastoral Agriculture Science and Technology, Lanzhou University, Lanzhou 730020, P.R. China; Curtin University, AUSTRALIA

## Abstract

The Qinghai–Tibetan Plateau is a very large land unit and an important terrestrial ecosystem within the Eurasian continent. Because of the harsh climate associated with the high altitude, alpine meadows on the plateau are susceptible to degradation from overgrazing. For this region, and for other alpine meadow pastures internationally, there is a need to define the sustainable stocking rate, to develop sound policy pertaining to future land use. Here we report biomass and liveweight gain per animal and per ha for pastures grazed by yaks at high, medium, or low stocking rates over 4 growing seasons from 2010 to 2013. Measures of herbage nutritive value are reported. The influence of inter-year variation in precipitation on standing herbage biomass was also evaluated. Higher precipitation increased standing herbage biomass and herbage nutritive value, indicating that vegetation suffered summer water deficit even in this environment. The sustainable stocking rate in this environment was determined to be approximately 1 yak ha^-1^ (grown from 80 kg to 120 kg liveweight in 90 d). At this stocking rate, yak weight gain per ha was 88% of that achieved at higher stocking rates typically used by farmers, but with little or no evidence of land degradation.

## Introduction

The Qinghai–Tibetan Plateau grassland is the largest geomorphological unit and one of the most important terrestrial ecosystems on the Eurasian continent [1]. Historically, this area has been grazed mainly by the Tibetan yak (*Bos grunniens*) and Tibetan sheep (*Ovis aries*). Global environmental changes are strongly affecting ecosystem functioning, and therefore, are affecting societies that rely on ecosystem services for subsistence [2].

The increase in the size of the human population has led to a greater pressure on land resources and demand for milk products and meat for human consumption, and has resulted in increases in stocking rates to levels that exceed the carrying capacity of the ecosystem [3]. Since the 1980s, increasing stocking rate has resulted in a substantial reduction in soil cover, which has led to degradation throughout much of the Tibetan Plateau grassland [4]. Surveys by the Chinese government and ecologists [5–9] have established that most of the alpine meadow ecosystem is overgrazed at present, and that this ecosystem on the Qinghai–Tibetan Plateau shows considerable signs of degradation.

Grassland degradation not only affects the resilience of ecosystems, but also jeopardizes the basic subsistence of dependent herdsmen. Overgrazing reduces herbage production [10] and alters species composition [11], and these changes in turn affect the productivity of grazing animals [12]. Furthermore, grazing directly affects litter deposition and the nutritive value of herbage [13], and indirectly affects vegetation community structure and composition. Under short-term grazing, herbage showed a significant improvement in nutritive value [14], which may have resulted from a change in community species composition [15]. However, under long-term overgrazing, there can be a shift from high- to poor-quality herbage species [16–18]. Consequently, both grassland ecosystem functioning and animal production are closely linked to damage from long-term overgrazing.

Overgrazing on the Qinghai–Tibetan Plateau is extremely common because the local grazing system aims to maximize animal liveweight gains per hectare (ha), which may produce higher economic returns in the short term. It has been reported that the local regulation of grazing activities of Tibetan Plateau yak herdsmen does not meet the targets for sustainable grassland-based animal production, from the view point of either ecology or economics [19]. Variations in rainfall directly affect grassland production, and indirectly affect the livestock carrying capacity of grassland ecosystems [20]. To establish rational management patterns, it is important to develop and define predictive models for rainfall variation, livestock carrying capacities, and biomass and nutrition production.

The current management pattern of herdmen of continuously grazing the same grassland area each year with high stocking rates, combined with variation in precipitation, makes grasslands more susceptible to degradation [21]. This poor strategy jeopardizes not only the stability and performance of the grassland ecosystem, but also the sustainability and productivity of the dependent animal production systems. Nevertheless, there have been few long-term surveys on the relationship between herbage productivity and livestock growth in this unique grazing ecosystem. Further research is needed to establish and standardize management systems for sustainable, grassland-based yak herding.

The focus of this study was to examine how different stocking rates in the Tibetan Plateau grasslands affect yak performance and vegetation, as reflected by changes in the nutritive value and yield of herbage. Specifically, the aim of the present study was to determine the effects of stocking rate and precipitation on the biomass, nutritive value, and nutritive yield of herbage, as well as on yak performance measured as liveweight gain (LG) per yak and per ha. The study also aimed to determine the extent to which the ecosystem is either damaged or improved in terms of its productivity and quality under different stocking rates and precipitation regimes. The interaction between herbage and yak stocking rate was analyzed to determine how LG responds to increasing stocking rate, and whether the expected grazing-induced increase in herbage nutritive values compensate for the expected decrease in biomass. We conducted a restricted grazing experiment to analyze the effects of yak grazing at different intensities, from ungrazed to heavily grazed, over four consecutive years (2010–2013). Based on the results of this study, we recommend an optimal and sustainable level of grazing that avoids overgrazing and prevents further grassland degradation.

## Materials and Methods

### Study area

The study area is located in Nannigou Village, Zhuaxixiulong Town, Tianzhu County (N 37.158°/E 102.813°, 2900–3000 m a.s.l.), Gansu Province, on the northeast edge of the Tibetan Plateau. This grassland area has an alpine climate with mean annual precipitation of 416 mm (1968–2013) and mean temperature of 0.7°C. Approximately 80% of the annual precipitation coincides with highest temperatures in warm season (May–September). The evaporation rate is 1521 mm and snow cover is 107 d in one year. Data for long-term mean climate variables as obtained from the Wushaoling meteorological station. The growing season lasts for approximately 150 d from May to September. The grazing season is slightly shorter than the growing season because early season grazing is prohibited to protect the grassland. Due to climate conditions in the Qinghai-Tibetan plateau, herdsmen divide alpine meadows into warm (from June to September) and cold (from October to May) paddocks respectively; this experiment was conducted in a warm paddock. The perennial dominant species *Polygonum viviparum* and *Kobresia capillifolia* contribute more than 50% to the herbage biomass yield. *Stipa purpurea*, *Leontopodium nanum* and *Saussurea pumila* are common accompanying species, and toxic plants include *Stellera chamaejasme*, *Oxytropis coerulea*, and *Gentiana farreri*. Soils are classified as Calcic Chernozems [22]. The experimental area had been moderately to heavily grazed by yaks until 2008. Thereafter, swards were allowed to recover for 1 year before the experiment started in June 2010. In 2010, 2011, 2012, and 2013, the biomass ranged from 891–1243, 940–1151, 824–1861, and 885–1512 kg ha^-1^ of the experimental paddock in July before grazing commenced.

### Experimental design

The effects of yak grazing on herbage biomass, herbage nutritive value, and herbage nutritive yield, as well as on yak LG, were analyzed using an experiment with a randomized complete block design. Experimental grazing periods were consistent with the typical local grazing season and lasted from June to September. Animal welfare and experimental procedures were carried out in accordance with the Guide for the Care and Use of Laboratory Animals, Ministry of Science and Technology of China [23], and were approved by Care and Use of Animals Center of Lanzhou University. Every effort was made to minimize animal pain, suffering and distress and to reduce the number of animals used. The duration of the grazing period was 88 d (June 26 to September 21), 90 d (June 24 to September 21), 93 d (June 23 to September 23), and 94 d (June 23 to September 23) in 2010, 2011, 2012, and 2013, respectively. For each grazing level, grassland production, yak intake, and grazing effects were analyzed along a gradient of four stocking rates (Table 1): (1) high stocking rate (HS, 1.25 yak ha^-1^), (2) medium stocking rate (MS, 1.00 yak ha^-1^), (3) low stocking rate (LS, 0.75 yak ha^-1^) and (4) no grazing (C). These were repeated three times for each stocking rate to give a total of 12 plots. Each plot was 4 ha. Yak management followed traditional practice, with animals grazing through the day and returning to a barn overnight; grazing time was about 10 h day^-1^. The paddock was owned by a local farmer, who agreed to its use for this experimental study. There was no endangered or protected species within the paddock.

**Table 1:** Average herbage utilization rate and stocking rates in numbers of yak ha^-1^grazing season^-1^ assigned to herbage utilization rate target ranges (TR) and grazing-intensity classification.

Grazing-intensity classification	C	LS	MS	HS
Stocking rate (yak ha^-1^ grazing season^-1^)	0	0.75	1.00	1.25
Herbage utilization rate TR (%)	-	<30	30–50	>50
2010	-	29.05±0.21	43.52±0.68	59.82±1.98
2011	-	27.25±0.78	40.10±1.02	55.91±0.48
2012	-	23.52±0.68	38.58±1.38	66.85±1.32
2013	**-**	28.93±1.05	45.84±1.50	61.69±2.19

Data represent seasonal means(±s.e.).

### Plant material sampling

A quadrat survey was conducted each year in the middle of July, August, and September. Nine quadrats (1 m × 1 m) were randomly placed in each plot, replicated three times for each stocking rate, ensuring that the distance to the edge of the plot was greater than 20 m. This gave a total of 1296 quadrats in four years. Plant species, height, and coverage were recorded within each quadrat. The plant samples from each quadrat were dried to constant weight at 85°C and then weighed. The samples were grounded in a mill through 0.25 mm sieve to minimize variation in nutritive values and yield arising from the effects of selective feeding by livestock at different sampling locations.

### Plant analysis and calculated indexes

Samples were analyzed in the laboratory to determine the concentration of nitrogen (N) and crude fat (CF) using AOAC methods [24]. The concentrations of neutral detergent fiber (NDF) and acid detergent fiber (ADF) were determined according to the method of Van Soest [25]. The amount of total digestible nutrients (TDN) was calculated using the following equation described by Horrocks [26]: TDN = (-1.291 × ADF) + 101.35; where ADF represents each of the digestible nutrient components. The metabolizable energy (ME; MJ kg^-1^) was calculated as follows: ME = TDN × 0.82 × 0.04409 × 4.182.

### Animal liveweight

The grazing animals were 16-month-old non-pregnant female yaks. All animals had free access to water and mineral lick stones and were given anthelmintic treatments at the beginning of June and the end of July each year, and shelters were provided. The average yak liveweight was 78, 77, 76, and 78 kg at the beginning of the grazing season in 2010, 2011, 2012, and 2013, respectively. According to yaks liveweight and intake, a standard yak with 200 kg equals to 4 sheep equivalents. The yaks in each plot had similar initial live weights, which were determined after a 15-d adaptation period. Yaks were weighed on two consecutive days, and the liveweight for each animal was calculated as the average of these two measurements. Seasonal LG per yak (LG yak) in g yak^-1^ d^-1^ (1) and seasonal LG per ha (LG ha) in g ha^-1^ d^-1^ (2) were calculated as follows:

$$LG_{yak}=\frac{\left(L_{E}-L_{B}\right)}{d}$$ (1)

$$LG_{ha}=LG_{yak}\times SR$$ (2)

where L_*i*_ is the mean liveweight of yak in each treatment (g yak^-1^) at the beginning (B) and end (E) of the grazing season, d is the number of grazing days, and SR is the stocking rate (yak ha^-1^ grazing season^-1^).

### Statistical methods

Data were analyzed by ANOVA using the Mixed Model option in SPSS version 16.0 (SPSS Inc. Chicago, IL, USA) with an autoregressive covariance structure. “Stocking rate”, “block”, “year” and their interactions were considered fixed effects with “year” treated as a repeated effect and “stocking rate × block” as the subject effect. Multiple comparisons of means were made by Tukey’s test [27]. Statistical significance was tested at the 0.05 level of probability. Simple linear and quadratic regression analyses in SPSS were used to analyze relationships between herbage biomass and precipitation, herbage biomass and stocking rate, herbage nutritive value and precipitation, herbage nutritive value and stocking rate, and animals and stocking rate.

## Results

### Precipitation

**Fig 1:**
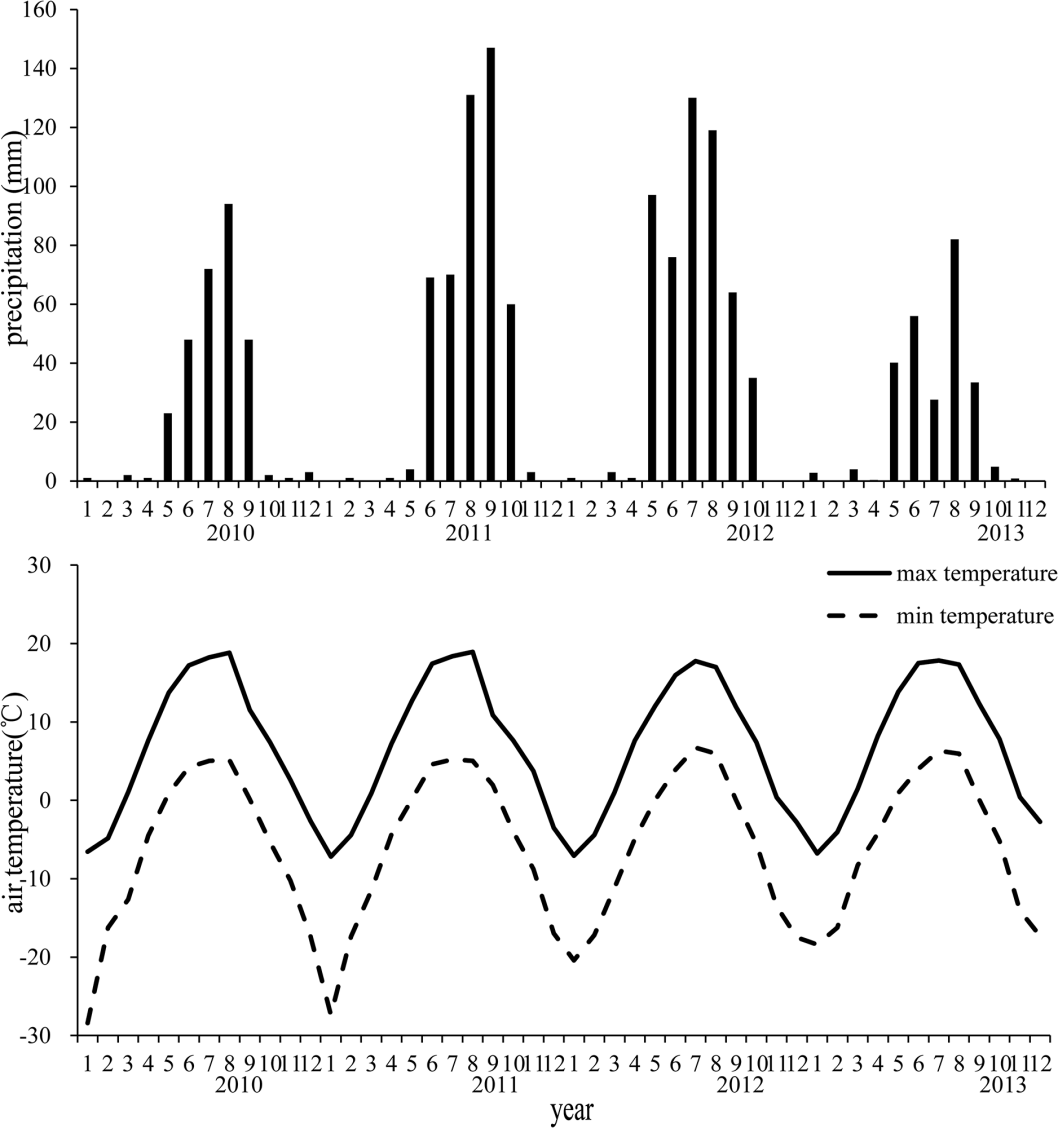
Mean monthly air temperature and monthly precipitation from 2010 to 2013 at the experimental site (37.16N, 102.81°E), located in an alpine meadow, Qinghai–Tibetan Plateau.

Annual precipitation was 295, 486, 526, and 252 mm in 2010, 2011, 2012, and 2013, respectively. During the experimental period (2010–2013), average annual precipitation was 390 mm, the variation in annual precipitation was 33% (Fig 1). The precipitation during the growing season from May to September was 231, 423, 486, and 221 mm in 2010, 2011, 2012, and 2013, respectively. Therefore, 78%, 87%, 92%, and 88% of the annual precipitation occurred during the growing season in 2010, 2011, 2012, and 2013, respectively. The long-term average annual precipitation is 416 mm, thus, there were drier conditions in 2010 and 2013, average conditions in 2011, and wetter conditions in 2012.

### Herbage biomass

**Fig 2:**
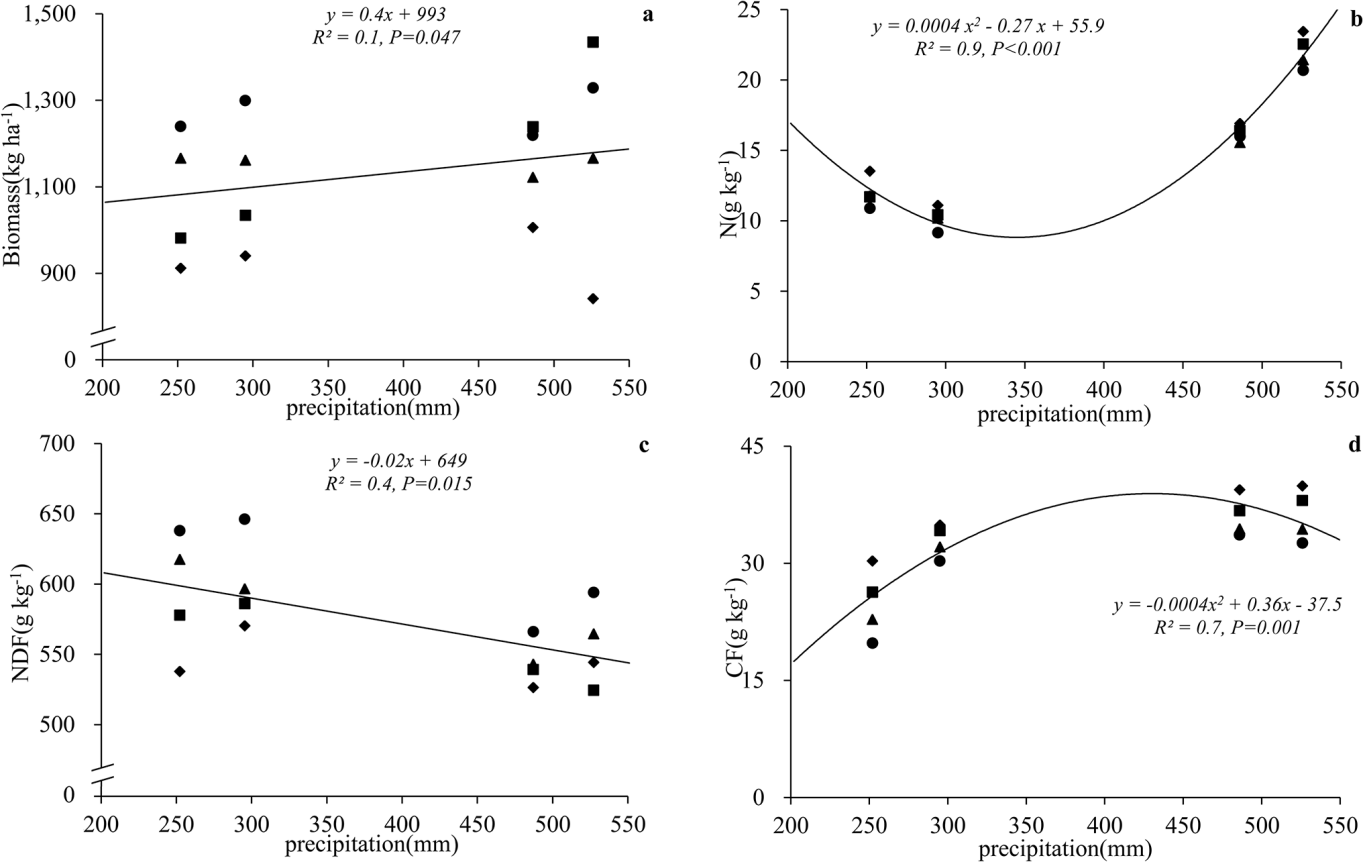
Relationships between (a) annual precipitation (mm) and herbage biomass (HB, kg ha^-1^) and between annual precipitation and herbage nutritive value parameters (g kg^-1^), as (b) nitrogen (N), (c) neutral detergent fiber (NDF) and (d) crude fat (CF). **Circles (●), triangles(▲), squares(■)and rhombuses(◆)represent no grazing (C), low stocking rate (LS), medium stocking rate (MS) and high stocking rate (HS), respectively.** Study site was located in an alpine meadow, Qinghai–Tibetan Plateau.

Herbage biomass was greater in years with higher precipitation. Averaged over all stocking rates, standing herbage biomass was 1109, 1147, 1193, and 1075 kg ha^-1^, in seasons with recorded precipitation rates of 295, 486, 526, and 252 mm in 2010, 2011, 2012, and 2013, respectively. Regression analysis defined a positive linear relationship between precipitation and standing herbage biomass (F = 19.6, df = 3, error df = 12, R^2^ = 0.9, *P* = 0.047) (Fig 2).

**Fig 3:**
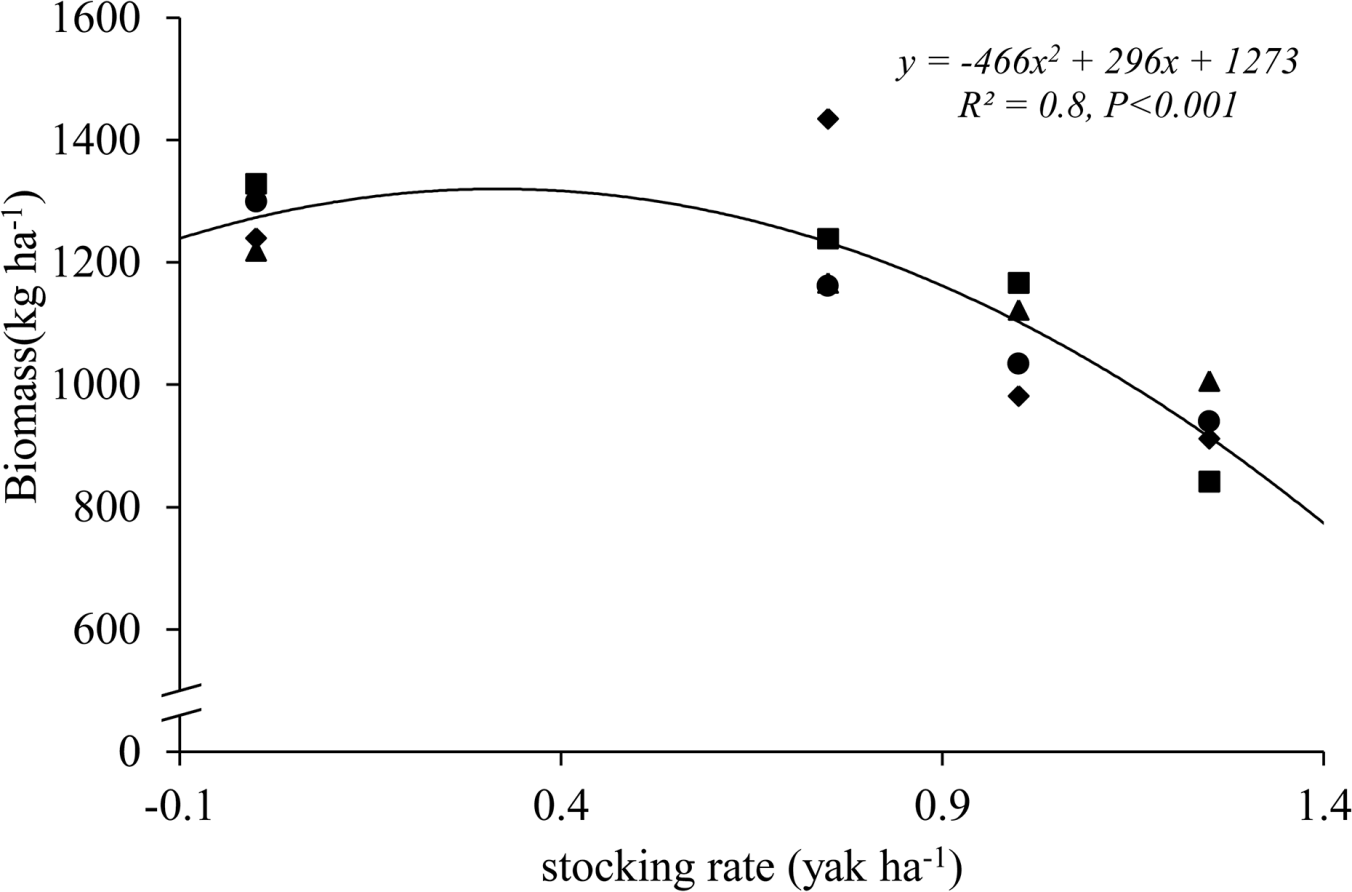
Relationship between stocking rate (i.e. stocking rate in yak ha^-1^ grazing season^-1^) and herbage biomass (kg ha^-1^). Circles (●), triangles(▲), squares(■)and rhombuses (◆)represent the 2010, 2011, 2012 and 2013, respectively. Study site was located in an alpine meadow, Qinghai–Tibetan Plateau.

Stocking rate affected many of the herbage parameters (Table 2), and had the strongest negative quadratic effect on herbage biomass (F = 21.1, df = 3, error df = 12, R^2^ = 0.8, *P*<0.001) (Fig 3). Herbage biomass was higher in C (1272 kg ha^-1^) than in HS (925 kg ha^-1^). Herbage biomass reduction associated with grazing by yaks was 0%, 2%, 15%, and 27% in C, LS, MS, and HS, respectively. There was a negative quadratic (F = 21.1, df = 3, error df = 12, R^2^ = 0.8, *P*<0.001) more than a negative linear (F = 17.1, df = 3, error df = 12, R^2^ = 0.6, *P* = 0.019) relationship between herbage biomass and stocking rate (Fig 3).

**Table 2:** Effects of stocking rate on herbage attributes and liveweight gain of yaks in an alpine meadow on the Qinghai-Tibetan Plateau.

Variable	Stocking rate					P-values of variables				
df						Y	B	SR	Y×SR	Y×B
	C	LS	MS	HS	s.e.	3	2	3	9	6
HB (kg ha^-1^)	1271a	1250a	1076b	924c	19	0.047	0.116	<0.001	0.037	0.390
N (g kg^-1^)	14.2a	14.8a	15.3a	16.3a	0.5	<0.001	0.844	0.046	0.999	0.997
CF (g kg^-1^)	29c	31bc	34ab	36a	6.2	0.001	0.660	0.029	0.886	0.974
ADF (g kg^-1^)	331a	317ab	301bc	281c	38	0.693	0.461	0.021	0.777	0.800
NDF (g kg^-1^)	611a	580ab	558bc	546c	65	0.015	0.739	0.011	0.835	0.955
TDN (g kg^-1^)	59b	60b	64a	64a	0.5	0.693	0.461	0.037	0.437	0.800
ME (MJ kg^-1^)	8.9b	9.1b	9.6a	9.7a	0.1	0.692	0.462	0.037	0.434	0.803
LG (g yak^-1^ d^-1^)	-	489a	439b	394c	5.4	<0.001	0.026	0.017	0.297	0.917
LG (g ha^-1^ d^-1^)	-	367c	440 b	493a	10	<0.001	0.055	0.001	0.016	0.991

Notes: biomass (HB), nitrogen (N), crude fat (CF), neutral detergent fiber (NDF), acid detergent fiber (ADF), total digestible nutrients (TDN), metabolizable energy (ME), liveweight gain (LG) per yak and per ha.

### Herbage nutritive value

Precipitation and stocking rate strongly affected herbage nutritive values (Table 2). The inter-year variation in the nutritive concentrations of the herbage corresponded closely with the annual precipitation rates, i.e., N concentrations in herbage were lowest in 2013 (252 mm precipitation) and highest in 2012 (526 mm precipitation), had a negative quadratic (F = 162.9, df = 3, error df = 12, R^2^ = 0.9, *P*<0.001) more than a negative linear relationship (F = 53.3, df = 3, error df = 12, R^2^ = 0.8, *P* = 0.008) with precipitation (Fig 2). The lowest herbage CF concentrations and the highest NDF concentrations were in 2013, a drier year (Fig 2). TDN and ME was not significantly related to precipitation.

**Fig 4:**
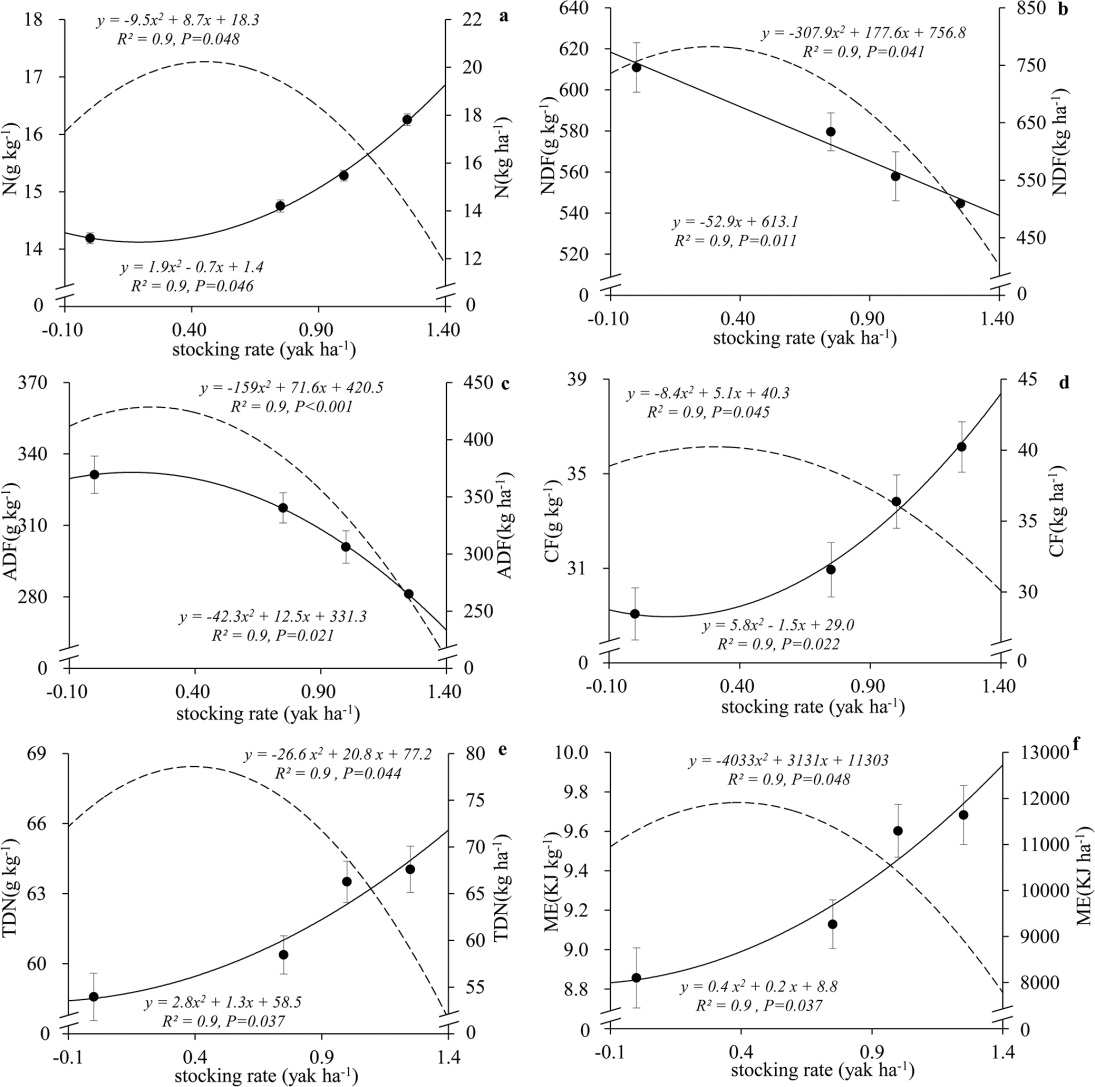
Relationships between stocking rate and herbage nutritive values (left y-axis, g kg^-1^) and herbage nutritive yields (right y-axis, kg ha^-1^): (a) nitrogen (N), (b) neutral detergent fiber (NDF), (c) acid detergent fiber (ADF), (d) crude fat (CF), (e) total digestible nutrients (TDN), and (f) metabolizable energy (ME). Solid lines show concentrations (●), and long-dashed regression lines show yields of herbage nutritional parameters (yields = concentrations × HB). Study site was located in an alpine meadow, Qinghai–Tibetan Plateau.

The nutritive value of herbage improved with increasing stocking rate (Table 2), that is, the concentrations of fiber fractions NDF and ADF decreased, while those of N, TDN, and ME increased with increasing stocking rate. These effects are illustrated in Fig 4, which highlights the linear relationships between stocking rate and NDF, and a quadratic relationship between stocking rate and N, ADF, CF, TDN, and ME. The nutritive yields were also analyzed by regression analysis, with N, CF, TDN, and ME yields showing inverse patterns with respect to their respective concentrations (Fig 4). The concentrations of TDN and ME significantly increased with NDF decline (*P*_*TDN*_ = 0.011; *P*_*ME*_ = 0.010).

### Animal liveweight gain

**Fig 5:**
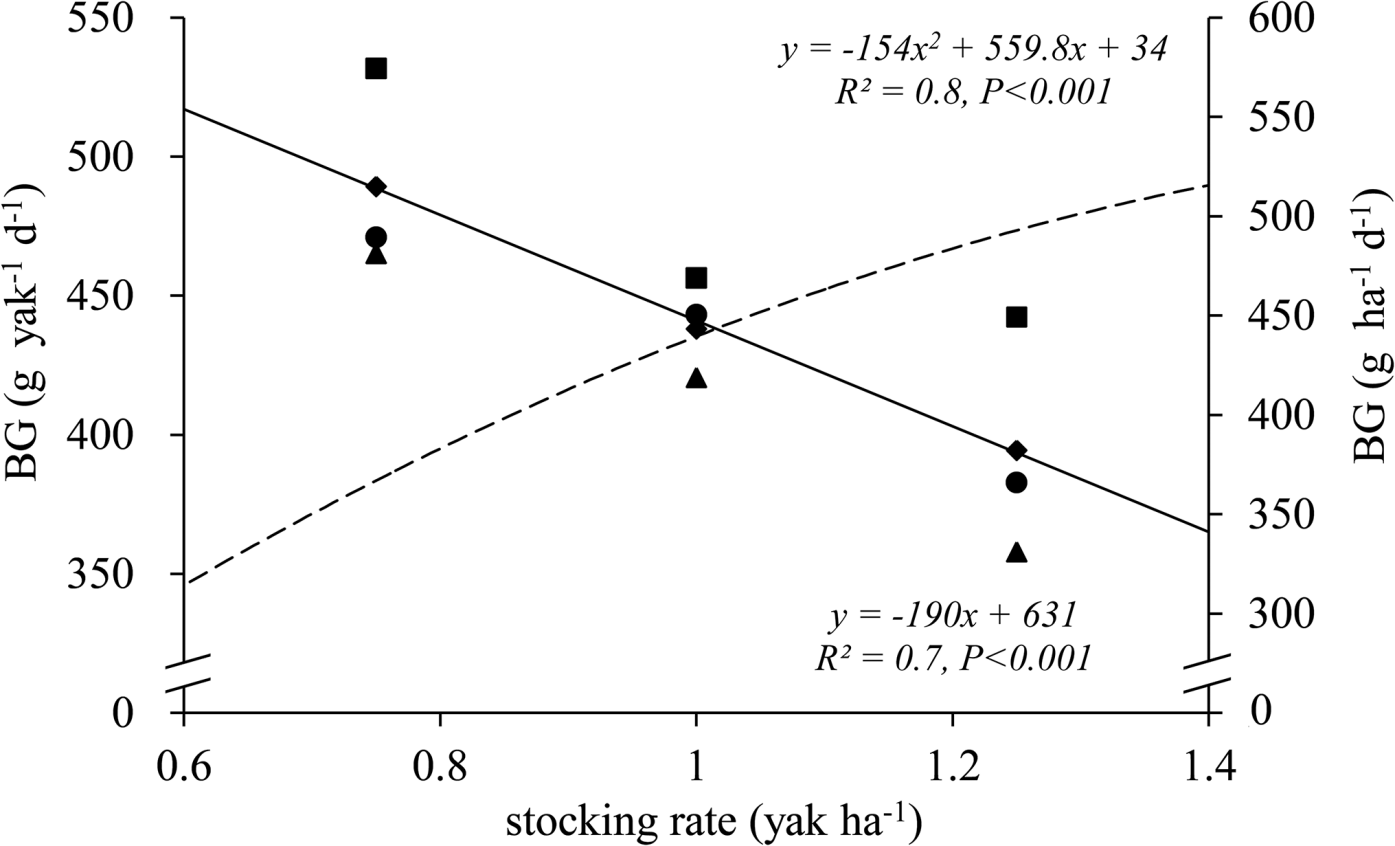
Relationships between stocking rate (i.e. stocking rate in yaks ha^-1^ grazing season^-1^) and liveweight gain (LG) of yaks (left y-axis, g yak^-1^ d^-1^) and area-based LG of yaks (right y-axis, g ha^-1^ d^-1^). Solid lines show LG per yak (g yak^**-1**^ d^**-1**^), and long-dashed regression lines show LG per ha (concentrations × SR, g ha^**-1**^ d^**-1**^).Circles (●), triangles(▲), squares(■)and rhombuses (◆)represent the 2010, 2011, 2012 and 2013, respectively. Study site was located in an alpine meadow, Qinghai–Tibetan Plateau.

Precipitation significantly affected yak LG (Table 2). In 2010, 2011, 2012, and 2013, the average LG was 432, 415, 477, and 441 g yak^-1^ d^-1^ and 425, 405, 469, and 433 g ha^-1^ d^-1^, respectively. Therefore, maximum animal performance and productivity was in the wet year, 2012. However, stocking rate was the most important factor in determining LG per yak (F = 1336, df = 3, error df = 24, R^2^ = 0.9, *P* = 0.017) and per ha (F = 17.8, df = 3, error df = 24, R^2^ = 0.8, *P* = 0.001). The daily LG per yak was significantly higher in LS than in MS and HS (Table 2). In contrast, the LG per ha increased with increasing stocking rate, with the maximum LG per ha in HS. The year × stocking rate interaction for LG per ha was statistically significant (F = 3.71, df = 6, error df = 96, *P* = 0.016) (Table 2). Therefore, inter-year differences in LG per yak were significant when comparing 2012 and other years in the three grazing treatments. LG per yak responded similarly to stocking rate in all four years, with the highest values in LS. In all four years, the lowest values for LG per yak were in HS (Fig 5). The interactive effect of year ×stocking rate was supported by the results of the regression analysis. The maximum LG per yak was in LS in 2012. The regression analysis also showed inter-year differences in the relationship between LG per ha and stocking rate. Grazing-induced decreases in individual LG resulted in diminishing responses of LG per ha, as described by a significant negative linear correlation. The maximum LG per ha was in HS in 2011 (stocking rate 1.25 yak ha^-1^). The function among stocking rate, biomass and animal production ha^-1^ was as LG_ha_ = 162+ 259SR+ 0.0114HB (R^2^ = 0.4, *P*<0.001), then LG_yak_ = 599- 178SR+ 0.0184HB (R^2^ = 0.3, *P*<0.001) and was used to describe the relationship among SR, biomass and animal production per head.

## Discussion

### Herbage biomass

Our results indicate that inter-annual variability in precipitation can explain the inter-year differences in herbage biomass. The standing biomass of herbage increased linearly with increasing annual precipitation. Because of the high variability in precipitation, herbage biomass varied markedly among the four years. Such a situation makes it difficult to predict the best stocking rate in any single year, which has led to the excessive stocking rate in this region. A yak growing from 80 kg to 120 kg would need around 360 kg DM biomass (40 MJ/d energy intake, and feed was about 9.5 MJ/kg in this study) per yak for a 90 day grazing season, then, we could image the utilization ratio of standing biomass is 33.5% in dry year (2013) and 30.2% in wet year (2012).

The results of this study also demonstrate that stocking rate adversely affected herbage biomass. The herbage biomass after grazing, which was a useful indicator of whether herbage biomass production was adequate to support the applied stocking rate, indicated that the gradations in stocking rate were appropriately chosen. Herbage biomass showed a negative linear relationship with stocking rate; at the end of the four years, there was 27% lower herbage biomass in the high stocking rate (HS) treatment than in the ungrazed treatment (C). This appears to be a common herbage response in the alpine meadow ecosystem, at least in the short term, because comparatively small biomass reductions in successive years have a cumulative effect. There is an ongoing argument as to whether grazing at current stocking rates will increase or decrease the primary productivity of grasslands [28, 29].

There is no intrinsic contradiction between the results of our study and those of other studies [30, 31]. Our research results are influenced by several factors. The relationship between herbage and animals is complex and strongly affected by many factors such as the duration and intensity of grazing, plant species composition and the type of grazing animals. Any differences between our results and those of other studies[11, 32, 33] may be related to yak being grazed approximately 90 days, much shorter than other animals like cattle or sheep (at least 6 months), and the stocking rates was 0.75–1.25 yak ha^-1^, lower than high-productive grassland (more than 10 sheep ha^-1^).

### Herbage nutritive values

High inter-year variability in precipitation explained most of the inter-annual variation in herbage nutritive values. Thus, not only the grassland biomass but also the nutritive values of herbage depended on the amount of precipitation. Our results suggest that herbage nutritive values increased with higher rainfall. In alpine meadows, precipitation directly determines the amounts of plant-absorbable water and available water. Because of accelerated mineralization, an increase in precipitation is associated with an increase in the amount of plant-available nitrogen. However, while higher moisture levels increase nitrogen availability, they also increase the nitrogen assimilation rate of plants. There are several explanations for the increase in nitrogen assimilation with increasing precipitation, including the increased activities of enzymes involved in nitrogen anabolism [34], increased net photosynthetic rate [35], and increased stomatal conductance [36].

The herbage contained more fiber in the drier year, 2013, than in the other years. Plant maturation was accelerated by the drier in this year. Thus, water stress resulted in more fibrous, less digestible herbage, whereas higher precipitation rates increased herbage nutritive values, likely because of delayed maturation. Plant maturation can be delayed under moderate water stress, and so herbage nutritive values remain at higher levels [37]. The lower herbage biomass in the drier year resulted in less accumulated litter. In the post-drier year, the low proportion of litter in the grassland resulteed in relatively high nutritive values was reported [38].

Our result that herbage nutritive values increased with increasing stocking rate is consistent with the results of other studies [39–41]. The herbage nutritive values correlation index (R) that related precipitation and stocking rate ranged from 0.1 to 0.7 and 0.8 to 0.9, respectively, so stocking rate has a larger effect than precipitation on herbage nutritive values. There are several explanations for this finding. The plant tissues have a lower mean age under heavy grazing than under light grazing, and the regrowing plant tissues have higher nitrogen content but lower fiber content [32]. However, the leaf residence time is permanently decreased under long-term intensive grazing, resulting in less or even no plant material reach the next phenological stage, and so less organic matter is fed back into the soil. As reported by Fanselow [42], a large proportion of the nitrogen reserves in stems and roots is mobilized to new shoots and leaves in grazed grass. Also, the decrease in herbage biomass caused by grazing can increase the nitrogen supply to remaining leaves, because of the higher relative nitrogen uptake rate of grazed grass [43]. Other studies have also reported positive effects of grazing on nitrogen concentrations in herbage, as a result of nitrogen inputs from the dung and urine of grazing animals [44]. Herbivore excretions typically accelerate the mineralization rates of senescent plant litter on the soil surface. Subsequently, the mineral nitrogen increases plant productivity. However, our results indicated that grazing negatively affected nitrogen yield, as seen from the inverse trends of nitrogen concentrations and nitrogen yields with respect to grazing (Fig 4A). The decrease in herbage biomass counterbalances the positive effect of grazing on nitrogen concentrations. Because CF is correlated with nitrogen content [45], CF showed the same trend as nitrogen(Fig 4D). Therefore, nutritive yields depend on herbage biomass, rather than nutritive values.

The results of this study indicate that grazing positively affected herbage nutritive values over the four-year short-term trial. However, long-term heavy grazing may negatively affect herbage nutritive values in alpine meadows, because there can be a shift from high- to poor-quality herbage species after long-term grazing [46, 47]. The change in herbage species under intensive grazing can also include invading weeds and less-preferred species. Our results suggest that the typical changes in the species composition of the Qinghai–Tibetan Plateau alpine meadow under long-term grazing are characterized by a shift from palatable perennial, such as *P*. *viviparum* or *K*. *capillifolia*, to unpalatable species (e.g. *O*. *coerulea* or *S*. *chamaejasme*), annual species (e.g. *Artemisia annua*), and subshrubs (e.g. *Potentilla fruticosa*).

### Animal liveweight gain

Our results show that variations in precipitation among years affected LG per yak and per ha, as reported in other studies [48, 49]. In the drier year of 2010, animal LG was lower than that in the post-drier years because of reduced availability and lower nutritive value of herbage. The interactive effect of year ×stocking rate on LG per yak shows that the optimum stocking rate to maximize LG was consistent among years with different precipitation rates (Fig 5). Therefore, the maximum LG per yak was achieved under the lowest stocking rate. However, the LG per yak under heavy grazing in 2012 was higher than those under lower stocking rates in other years. From that, we conclude that wet years permit more intensive grazing than do dry years (Fig 5). Because heavy grazing reduces herbage biomass, yaks must graze for longer per day, or increase their intake, to compensate for the decreased biomass and availability of herbage [50, 51]. Extending the grazing period results in an increase in energy expenditure during grazing and trampling movements, which explains the lower individual LG of yaks in HS than in C (Table 2, Fig 5). Increased feed intake relies on augmenting herbage biomass [52], because an increased amount of herbage enables yaks to graze more selectively. Yaks will select not only more palatable species, but also more palatable parts of plants such as the upper layers of grass leaves. There are higher concentrations of N and CF and less fiber in upper leaves than in lower parts of leaves and stems [53]. The relationship between LG and herbage biomass showed that LG is determined by herbage biomass, that is, yak LG was lower when there were lower levels of herbage biomass (Fig 5).

The stocking rate partly determines the profitability and productivity of the animal grazing ecosystem. For example, high stocking rate leads to maximum LG per ha, resulting in higher profits in the short term. The results of our study indicate that intensive grazing (HS) can reduce herbage biomass by 27% (Table 2). We found that LG per ha positively correlated to both biomass and stocking rate, however LG per yak positively correlated biomass, but negatively correlated with stocking rate. Herbage biomass reduction caused by grazing not only alters species composition, but also reduces soil cover [54], which leads to increases in wind and water erosion that further degrade these alpine meadows [55]. The recommended SR in the region targets the health grasslands (biological optimum), and benefits herdsman’s livelihood (economic optimum) as well. Therefore, HS may provide the highest LG per ha in the short term, but it jeopardizes the profitability and ecological functioning of the grassland livestock system in the longer term [56]. We suggest that a sustainable stocking rate should not exceed MS, with a stocking rate of 1 yak ha^-1^ grazing season^-1^. Grazing at this intensity avoided the reductions in herbage biomass typically associated with grazing, and the LG per ha was 88% of that obtained in HS (Table 2). This result is similar to that of Kemp and Michalk [57], who argued that the optimal stocking rate of grassland was one that satisfies production goals and protects the environment. An optimal stocking rate should assure the sustainable development of grassland and livestock management. To meet these goals, the stocking rate should be reduced to MS.

## Conclusions

Herbage biomass and herbage nutritive values were dependent on annual precipitation. Grazing also affected herbage performance, in that herbage biomass decreased and herbage nutritive values increased with increasing stocking rate. The increase in herbage nutritive values by grazing could not compensate for the decrease in herbage biomass. Both precipitation and stocking rate were important factors in determining livestock LG. We conclude from our results that a moderate stocking rate of 1 yak ha^-1^, which provides 88% of the highest LG per ha, satisfies the demands for sustainable grazing management. However, other more developed grazing management strategies, such as rotation grazing, supplementary feeding in winter and yak turn over period from 4 years to 2 years, may also increase the biomass and nutritive value of herbage and animal LG in the winter. Implementation of such strategies may improve the sustainability of grassland use on the other parts of Qinghai-Tibetan Plateau with the similar conditions.
